# Molecular Characteristics of Chicken Infectious Anemia Virus in Central and Eastern China from 2020 to 2022

**DOI:** 10.3390/ani13172709

**Published:** 2023-08-25

**Authors:** Shuqi Xu, Zhibin Zhang, Xin Xu, Jun Ji, Lunguang Yao, Yunchao Kan, Qingmei Xie, Yingzuo Bi

**Affiliations:** 1 Henan Provincial Engineering and Technology Center of Health Products for Livestock and Poultry, Nanyang Normal University, Nanyang 473061, China; xushuqi0713@126.com (S.X.); zhangzhibin1018@163.com (Z.Z.); jzbetty@163.com (X.X.); lunguangyao@163.com (L.Y.); kanyunchao@163.com (Y.K.); 2 Henan Key Laboratory of Insect Biology in Funiu Mountain, Nanyang Normal University, Nanyang 473061, China; 3 College of Animal Science, South China Agricultural University, Guangzhou 510642, China; xqm0906@126.com (Q.X.); yingzuobi@163.com (Y.B.)

**Keywords:** chicken infectious anemia virus, sequence analysis, evolutionary analysis, recombination, mutation

## Abstract

**Simple Summary:**

An immune-suppressive disease known as chicken infectious anemia (CIA) develops after infection with chicken infectious anemia virus (CIAV). This study involved a systematic analysis of the epidemiology and genomics of CIAV in the provinces of Henan, Anhui, Jiangsu, and Hubei in China. The positive rates of the samples from each province flock ranged from 50% to 80%. Meanwhile, coinfections of CIAV with Marek’s disease virus, avian leukosis virus, infectious bursal disease virus, and fowl adenovirus were also identified. This study revealed the diversity of CIAV genomes as well as key mutation sites and intricate recombinants. The study findings underscore the importance of CIAV surveillance and provide a basis for further investigation into the evolution and molecular characteristics of CIAV strains.

**Abstract:**

To evaluate the recent evolution of CIAV in China, 43 flocks of chickens from the provinces of Henan, Jiangsu, Hubei, and Anhui were screened via polymerase chain reaction during 2020–2022. Of these, 27 flocks tested positive for CIAV nucleic acids, including 12 which were positive for other immunosuppression viruses. Additionally, 27 CIAV strains were isolated, and their whole genomes were sequenced. The AH2001 and JS2002 strains shared the highest identity at 99.56%, and the HB2102 and HB2101 strains shared the lowest identity at 95.34%. Based on the genome sequences of these strains and reference strains, a phylogenetic tree was constructed and divided into eight main branches. Most of the strains were grouped with the East Asian strains, whereas the HB2101 strain belonged to the Brazil and Argentina cluster. A recombination event was detected in multiple strains, in which AH2002 recombined from KJ728827/China/2014 (from Taiwan Province) and HN2203, and AH2202 recombined from KX811526/China/2017 (from Shandong Province) and HN2203. All the obtained strains had a highly pathogenic Gln amino acid site at position 394 of the VP1. Overall, our findings demonstrate the importance of CIAV monitoring and provide data that aid in understanding the evolution of CIAV.

## 1. Introduction

The viral disease known as chicken infectious anemia (CIA) is caused by the chicken infectious anemia virus (CIAV) and leads to significant economic loss to the poultry industry worldwide [1,2]. Young birds without maternal antibodies are highly susceptible to develop primary signs of CIA, which include anemia, subcutaneous hemorrhages, growth retardation, aberrant feathers, limb paralysis, thymus atrophy, and bone marrow atrophy. Infected birds are often immunocompromised, which enhances the risk of secondary viral, bacterial, or fungal infections, even though CIA is frequently regarded as asymptomatic in adult chickens [3].

CIAV was first isolated in Japan in 1979 and has since been found to spread both vertically and horizontally [4]. Horizontal transmission throughout the flock occurs via excrement, dander, or feathers, primarily through oral infection. Notably, as vertical infection disseminates through eggs, it is quite likely that an outbreak of CIA in the progeny will occur [5]. Additionally, previous research has shown that vertical transmission of CIA can occur even when infected hens have developed an immune response [6].

CIAV belongs to the Anelloviridae family and the Gyrovirus genus [7]. Hematopoietic cells and T-lymphocytes of chickens are the primary targets of this nonenveloped virus, which has icosahedral single-stranded DNA [8]. One circular and single-stranded DNA molecule with a covalently closed end constitutes the whole CIAV genome. The genome with a length of 2.3 kb codes for peptides with molecular weights of 51 (VP1), 28 (VP2), and 13.6 (VP3) kDa [9]. VP1 is the only structural protein formed in the CIAV capsid, and VP2 functions as a scaffold protein, assisting VP1 in forming the proper shape and exposing its epitope [10]. Both VP1 and VP2 contain epitopes that elicit a neutralizing antibody response [11]. VP2 is also involved in reducing virulence and triggering apoptosis [12]. The nonstructural protein VP3 (13 kDa) mainly promotes apoptosis in infected chicken cells [13,14].

In numerous areas of China, CIAV has been detected in poultry farms, and its isolation from specific pathogen-free chickens may explain its widespread dissemination and vaccine contamination [15]. Reticuloendotheliosis virus (REV), fowl adenovirus (FAdV), and avian leukosis virus (ALV) are common viruses that cause immunosuppressive infections and have been linked to CIAV in a growing number of instances [16,17]. Continuous investigation of CIAV is necessary to understand its complex evolution process, which involves complex recombination and extensive mutations [5,18].

To evaluate the recent evolution of CIAV in China, molecular characteristics of 27 CIAV were systematically analyzed on the basis of their genome sequences, including phylogenic mutation and recombination analysis. Our findings demonstrate the importance of continuous CIAV monitoring and provide data that can help in understanding the recent evolution of CIAV.

## 2. Materials and Methods

### 2.1. Sample Collection and Pathogen Detection

During 2020–2022, 430 clinical samples (pooled samples of bone marrow, spleen, and thymus) were obtained from dead chickens which exhibited symptoms of depression, anorexia, emaciation, growth retardation, or pale skin. These birds were obtained from 43 chicken flocks (10 chickens per poultry flock) from the Chinese provinces of Henan, Anhui, Jiangsu, and Hubei. All the samples were stored at a temperature of −80 °C.

### 2.2. Detection, Polymerase Chain Reaction (PCR) Amplification, and Sequencing

Following the manufacturer’s instructions, viral DNAs and RNAs were separately extracted from the chicken samples using a commercial kit (TIANamp Virus DNA/RNA Kit, Tiangen, Beijing, China). Three primer pairs, as described previously, were used for the detection and genome amplification of CIAV [19]. The first primer pair consisted of a forward primer (P1: GCATTCCGAGTGGTTACTATTCC) and a reverse primer (P2: CGTCTTGCCATCTTACAGTCTTAT), with a projected target-band of 842 bp. The second pair of primers consisted of a forward primer (P3: CGAGTACAGGGTAAGCGAGCTAAC) and a reverse primer (P4: TGCTATTCATGCAGCGGACTT), with a projected target-band of 990 bp. The last pair of primers included a forward primer (P5: GAAAATGAGACCCGACGAGCAACAG) and a reverse primer (P6: GATTCGTCCACTTTGACTTTCTGTG), with a projected amplicon size of 736 bp. Primer pairs (P3 and P4) were used for CIAV detection, and all three primer pairs were used for complete genome amplification (P1–P6).

Meanwhile, ALV (F: GGATGAGGTGACTAAGAAAG; R: CGAACCAAAGGTAACACACG), IBDV (F: ATGTGGCTGGAAGAGAATGG; R: GCCCTTTGAGACTTGCTACCT), FAdV (F: AAGTTCAGGCAGACGGTCGTG; R: TAGTGATGCCGGGACATCATGTCG), REV (F: GCCTTAGCCGCCATTGTA; R: CCAGCCAACACCACGAACA), and Marek’s disease virus (MDV) (F: AGAAACATGGGGCATAGACG; R: CTTGCAGGTGTATACCAGGG) were also detected via PCR assay, as previously described [16,20,21,22].

To perform PCR, 100 ng of DNA template, 0.2 mM of each dNTP, 2 mM of MgCl_2_, 0.6 M of each primer, and 0.75 U of PlatinumTM Taq DNA Polymerase were prepared in 1× supplied PCR buffer (Thermo Fisher Scientific Baltics UAB, Vilnius, Lithuania). Amplification was performed using the following conditions: the predenaturation step at 95 °C for 5 min, 35 cycles of the denaturation step at 95 °C for 30 s, the annealing step at 60 °C (53 °C for ALV; 55 °C for IBDV; 55 °C for FAdV; 52 °C for REV; and 56 °C for MDV) for 30 s, the extension step at 72 °C for 30 s (30 s for ALV; 25 s for IBDV; 45 s for FAdV; 50 s for REV; and 1 min for MDV), and a final extension step at 72 °C for 10 min. Both positive and negative controls were included, and the amplified products were subsequently separated via 1% agarose gel electrophoresis. The purified amplicons were cloned into a pMD18-T simple vector (TaKaRa Biotechnology Co., Ltd., Dalian, China) and sequenced (Hongxun, Suzhou, China). 

### 2.3. Virus Isolation

The CIAV present in the positive samples was cultured in MDCC–MSB1 lymphoblastoid cell lines following the previously described protocol [5]. Briefly, the homogenate suspension from the CIAV-positive samples was sterilized by filtration using a 0.22-μm filter and added onto the MDCC–MSB1 cells at 37 °C for 1 h. After virus absorption, the MSB1 cells were cultured in 5 mL of 1640 medium containing 2% fetal bovine serum (Gbico, New York, NY, USA) at 37 °C for 3 days. Five blind passages using MDCC-MSB1 cells were performed to enable viral growth. For each isolated CIAV, detection and genome amplification were performed using the PCR assays described above.

### 2.4. Phylogenetic and Recombination Analysis

Whole genome sequences of the 27 CIAV strains along with references were aligned and identified using the Lasergene 7.0 software (DNASTAR Inc., Madison, WI, USA). Next, an identity heatmap was created using an online program (https://www.chiplot.online/ (accessed on 26 May 2023)). In addition, the amino acid (aa) mutation sites of VP1 were summarized to assess genetic markers known to be associated with virulence.

The maximum likelihood method available in the molecular evolutionary genetics analysis (MEGA X) program was used with 1000 bootstrap replications to perform evolution analysis [23]. The online Evolview v3 program (http://www.evolgenius.info/evolview/ (accessed on 17 August 2023)) was used to visualize the generated phylogenetic tree [24].

To investigate the possibility of intergenic recombination, a total of 105 complete CIAV genomes from obtained and reference strains were analyzed. The Recombination Detection Program (RDP4), which includes the methods of GENECONV, BOOTSCAN, MaxChi, CHIMERA, SISCAN, and 3Seq, was used for the prediction of recombination events and the identification of potential parental sequences [25].

### 2.5. VP1 Structure Modeling

To visually reflect the VP1 structure change caused by the mutations in the CIAV isolates in this study, the tertiary structures of the VP1 of these isolates and the reference strain of SD24 were modeled using AlphaFold2.3.0 (https://wemol.wecomput.com/ui/#/ (accessed on 19 June 2023)).

## 3. Results

### 3.1. Sample Screening

**Figure 1:**
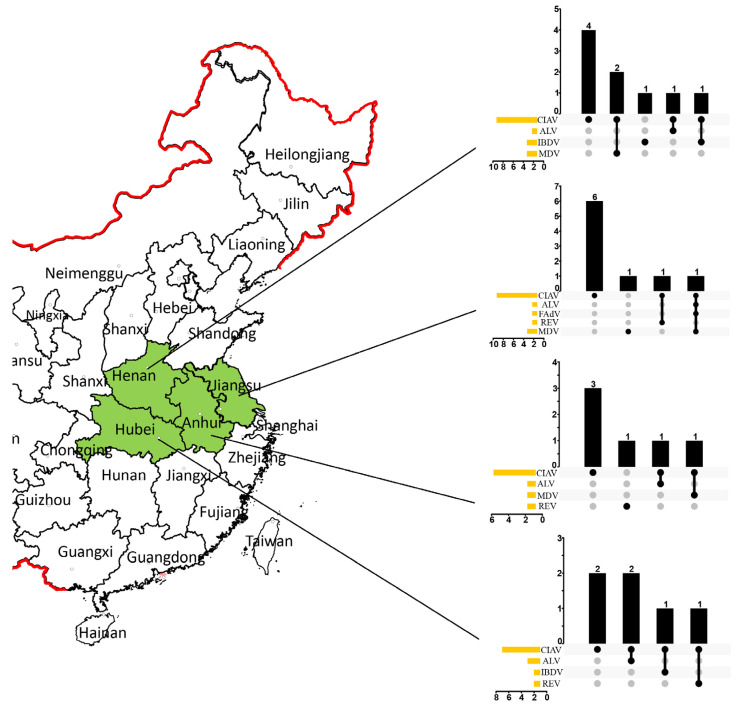
CIAV distribution in China and the coinfection status of ALV, IBDV, FAdV, REV, and MDV in various flocks. The chart marked with green represent the Province for CIAV investigation in this study. The UpSet plot presents the distribution of different viruses in the flocks. The bar chart above represents the number of positive flocks contained in each type of group, and the dotted line at the bottom right presents the infection status. The bar chart at the bottom left represents the number of positive numbers included in each type of virus.

Samples from 43 poultry farms were tested, and the results showed that 27 flocks were positive for CIAV, including 8 out of 13 (61.54%) from Jiangsu, 8 out of 12 (66.67%) from Henan Province, 6 out of 9 (66.67%) from Hubei, and 5 out of 9 (55.56%) from Anhui. The positive rates of the samples from each flock ranged from 50% to 80%, with an average of 69.3%. CIAV isolates were successfully obtained from positive samples in each flock. Among the 27 CIAV-positive flocks, 11 flocks of dual infections were caused by CIAV and the following coinfecting viruses: MDV (*n* = 3), REV (*n* = 2), ALV (*n* = 4), and IBDV (*n* = 2); additionally, one flock of quadruple infection was also identified (CIAV with FAdV + ALV + MDV). In CIAV-negative flocks, one flock each from Henan, Anhui, and Jiangsu was tested to be positive for IBDV, REV, and MDV, respectively. The infection and coinfection status of CIAV, ALV, IBDV, FAdV, REV, and MDV in various flocks was displayed via the UpSet map in Figure 1.

### 3.2. DNA Alignment and Identity Analysis

All 27 obtained CIAV strains had a complete genome length of 2298 bp, and the sequences were submitted to the GenBank database with accession numbers OQ869186–OQ869212. Viral sequences from positive samples and CIAV isolates from the same chicken flock shared 100% identical. Table 1 and Table 2 present detailed information on the 27 CIAV strains and 78 reference strains detected in chickens, humans, dogs, and cats. According to the sequence alignment of the entire genome, the nucleotide (nt) identity of the 27 CIAV strains ranged from 95.34% (HB2102 and HB2101) to 99.56% (AH2001 and JS2002). Between the 27 isolated and vaccine strains, HB2101 shared the lowest similarity with Cux-1 (95.39%) and Del-Ros (96.21%), whereas HN2101 had the highest similarity with Cux-1 (97.11%) and Del-Ros (98.3%). HB2102 and AF227982.1/Australia/2001 had the lowest similarity (91.97%), whereas HB1901 and KM496306.1/China/2013 had the highest similarity, (99.74%) compared with the reference strains. Supplementary Figure S1 illustrates the similarity comparison of all the strains. 

### 3.3. Phylogenetic and Recombination Analysis

**Figure 2:**
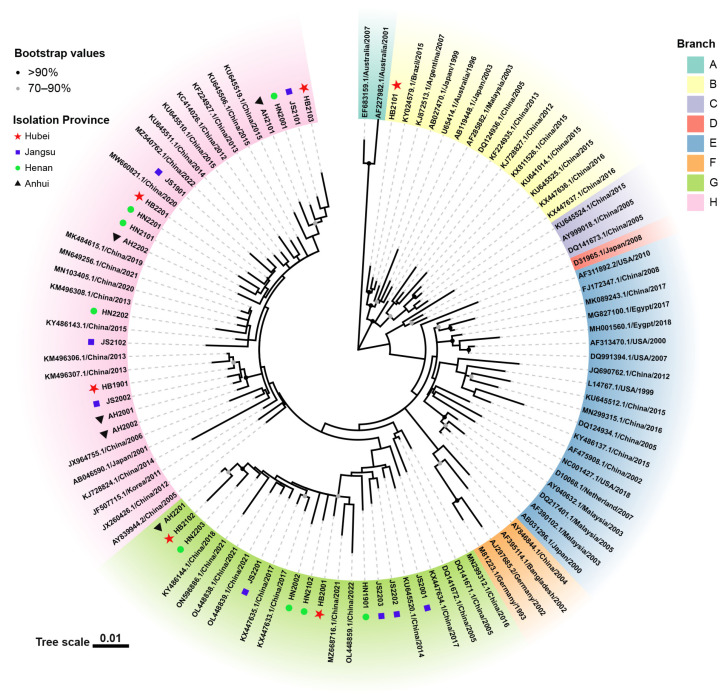
The CIAV phylogenetic tree based on the entire genome. In the ML analysis using MEGA X, the filled circles along the branches represent bootstrap values. The black triangle represents the strains from Anhui Province, the blue square the strains from Jiangsu Province, the green circle the strains from Henan Province, and the red pentagram the strains from Hubei Province.

The phylogenetic tree of the entire genome of the 27 obtained strains and 78 reference CIAV strains is illustrated in Figure 2. The evolutionary tree was separated into eight branches (A to H). HB2101 (Branch B) was clustered with KY024579.1/Brazil/2015 and KJ872513.1/Argentina/2007 in South America, whereas the other strains (Branch G and Branch H) more closely resembled Asian strains. Both vaccine strains (Del-Ros and Cux-1) that were widely used abroad were clustered into Branch E. Eleven of the 27 obtained strains (Branch G) solely resembled the Chinese strain, whereas the remaining 15 strains (Branch H) resembled the Korean (JF507715.1/Korea/2011) and Japanese (AB046590.1/Japan/2001) strains.

**Figure 3:**
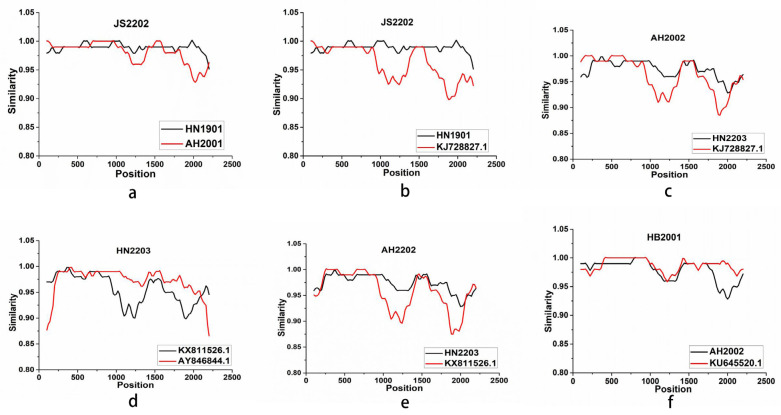
Recombination occurrence in JS2202, AH2002, HN2203, AH2202, and HB2001 strains analyzed using the Simplot software. (**a**) Recombination occurrence in JS2202. (**b**) Recombination occurrence in JS2202. (**c**) Recombination occurrence in AH2002. (**d**) Recombination occurrence in HN2203. (**e**) Recombination occurrence in AH2202. (**f**) Recombination occurrence in HB2001.

To assess the probability of genotype recombination, we analyzed the whole genomes of the 27 CIAV strains and the 78 reference strains using the RDP 4.83 and Simplot 3.51 software tools. The putative recombination event showed high confidence ratings and predicted five recombinant strains (JS2202, AH2002, HN2203, AH2202, and HB2001) on the basis of at least five independent detection methods (Table 3). Figure 3 illustrates the bootstrap analysis that we conducted to determine the breakpoint and identify each recombinant and its parent strain. Intriguingly, JS2202 may not only be formed by the recombination pair of major parent HN1901 and minor parent AH2001, but also by the recombination pair of major parent HN1901 and minor parent 18 (KJ728827.1/Taiwan/2014). Both the two recombinant events occurring in the JS2202 strain shared similar breakpoints from 89 to 1796 across the untranslated region (UTR, 2182 to 358), the VP2 coding region (359 to 1009), the VP3 coding region (465 to 830), and the VP1 coding region (832 to 2181). JS2202 shared similarity with HN1901 and AH2001 ranging from 0.93 to 1, and from 0.91 to 1 with KJ728827.1/China/2012.

### 3.4. Mutation Analysis of VP1

**Figure 4:**
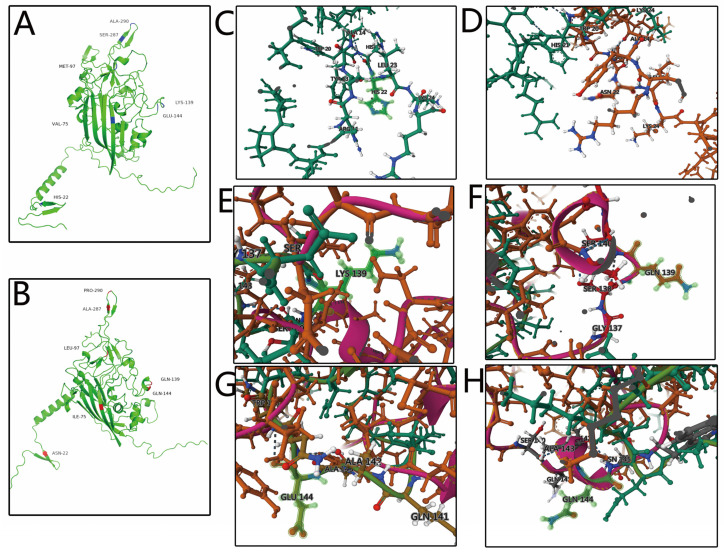
Cartoon scheme of the CIAV-VP1 protein structure. (**A**) Reference VP1 protein structure of strain SD24. (**B**) Mutant VP1 protein structure of strain HB2103. (**C**) Local protein structure of H22 of strain SD24. (**D**) Local protein structure of N22 of strain HB2103. (**E**) Local protein structure of K139 of strain SD24. (**F**) Local protein structure of Q139 of strain HB2103. (**G**) Local protein structure of E144 of strain SD24. (**H**) Local protein structure of Q144 of strain HB2103.

Previous studies have shown that aa position 394 is a major genetic determinant of pathogenesis, and sites 75, 89, 125, 139, 141, and 144 are related to viral replication and transmission [26,27,28]. In this study, Q394 was detected in all 27 strains that were analyzed, implying that these might be highly pathogenic strains [26]. T89 was found in all the analyzed strains; A89 was detected only in highly passaged CIAVs, but not sufficient to cause attenuation [27]. The 139 and 144 aa sites of HN2001, HB2101, JS2101, HB2103, and AH2101 contained the Q mutation, which is related to a reduced rate of cell-cultured spread [28]. Mutations at the important sites of VP1 are detailed in Table 4. Furthermore, the predicted tertiary structures of the SD24 and HB2103 strains indicated the structure change caused by mutations located at residues 22, 75, 139, and 144 (Figure 4).

## 4. Discussion

CIAV has been identified in chicken populations worldwide since it was originally discovered in China in 1996, posing a considerable threat to the poultry industry [29,30]. We screened 27 positive flocks in four provinces of China from 2020 to 2022, and the prevalence rates of CIAV were 61.54% (8/13) in Jiangsu province, 66.67% (8/12) in Henan Province, 66.67% (6/9) in Hubei Province, and 55.56% (5/9) in Anhui Province. These prevalence rates were higher than those reported in a survey of 13.30% of the average CIAV infection rate in 12 provinces from 2014 to 2015, indicating that CIAVs were more prevalent in recent years [31]. As previously reported, CIAV was mainly tested in a live attenuated vaccine against Newcastle disease, avian infectious bronchitis, and fowlpox [5]. Combined with vertical/horizontal transmission and contamination from live attenuated vaccine, CIAV has recently spread throughout the China and even globally, causing severe economic losses. Meanwhile, the coinfection of CIAV and other immunosuppressive viral agents such as ALV, REV, or IBDV might play a significant role in the infection course of CIAV. These results suggested the ongoing urgent need for continued screening tests for CIAV and other immunosuppression pathogens in clinical samples and vaccines.

The evolutionary tree of the 27 strains analyzed in this study and 78 reference strains revealed eight main branches, with the majority of the strains being closely related to the Chinese strains. However, the HB2101 strain showed a closer relationship to strains from Brazil and Argentina. The mechanisms and factors that contribute to the natural transmission of similar CIAV strains across borders remain to be determined. According to a previous study, local dissemination and long-distance migration are the main factors influencing CIAV evolution in South America. [32]. It is suspected that the transmission of the abroad-genotype CIAV is due to migratory bird movements or to the introduction of broiler breeders from abroad. These findings highlight the importance of detecting and monitoring the breeding chickens that are introduced as well as the migratory bird populations. Furthermore, the CIAV strains isolated in this study belonged mainly to Branch G and Branch H and were not locally determined, but distant from the internationally prevalent vaccine strains Cux-1 and Del-Ros, which belonged to Branch E. Although the genomic similarities between the 27 isolated and the two vaccine strains were almost similar, the different phylogenetic distribution suggested the distant-evolution trend.

Due to its genetic diversity, the VP1-enconding gene is commonly targeted for sequence analysis [33]. Several aa sites in VP1 are associated with viral pathogenicity and replication [9,26,27,28]. An important genetic predictor of virulence in CIAV is the aa at position 394, where pathogenic strains possessed glutamine (Q) whereas strains containing histidine (H) had a moderate virulence [26,28]. In this study, all 27 CIAV isolates carried Q394, indicating that they might be highly pathogenic. Cell culturing has demonstrated that aa located at 139 and 144 can influence viral growth and contagiousness and that CIAV strains harboring glutamine (Q) at these sites are less contagious [34]. Five (HN2001, HB2101, JS2101, HB2103, and AH21011) out of the 27 obtained strains had Q, indicating their weak contagiousness in cultured cells [34]. Meanwhile, amino acids at I75, L125, E141, and E144 of VP1 are related to reduced pathogenicity in chickens [9]. Further, A89 was detected in highly passaged CIAVs but was not sufficient to cause attenuation [27]. T89, I125, and Q141 were the major representative substitutions in VP1 of both genotype I and genotype II CIAV isolates in Egypt [35]. The CIAVs obtained in this study all harbored T89 and Q141, but the substitution 125 in VP1 was highly mutated. As shown in the predicted tertiary structures, mutations located at 22, 75, 139, and 144 may cause structure changes in VP1. These findings suggested the complicated relatedness between mutations sited in VP1 and CIAV pathogenicity. Given the genetic variability of VP1 and its crucial role in pathogenicity and transmissibility, the pathogenic differences between strains carrying extensive mutations require continuous and thorough evaluation.

Recombination is thought to be the main catalyst for viral evolution and the source of most viral mutations [36]. We discovered gene recombination events in five isolates (JS2202, AH2002, HN2203, AH2202, and HB2001). JS2202 may have resulted from a combination of the major parent HN1901 and the minor parent AH2001 as well as of the major parent HN1901 and the minor parent 18 (KJ728827.1/Taiwan/2014). The recombinant strain HN2003 and the strain 18 (KJ728827.1/Taiwan/2014) make up AH2002. Genetic recombination of CIAV has been observed to take place in the coding areas (nt 818–1295; nt 768–1286) [18,30], in the noncoding areas (nt 2108–2143; nt 2173–2231) [2,37], or in the regions that bridge the coding and noncoding regions (nt 1684–1757) [29]. The rearrangement breakpoint identified in this study spans both the coding and noncoding regions, extending from position 89 (the starting breakpoint) to position 1796 (the finishing breakpoint). Further investigations are necessary to trace the evolution of CIAV and establish the link between viral recombination and pathogenicity.

## 5. Conclusions

In conclusion, our study has revealed the complex infection status of CIAV in central and eastern China, the diversity of CIAV genomes, and key mutation sites and intricate recombinants. These findings provide a basis for further investigation into the evolution and molecular characterization of CIAV strains.

## Figures and Tables

**Table 1:** Detailed information regarding the reference stains used in this study.

Accession No.	Strain Name	Species	Site of Isolation	Genome Length (bp)	Year
M81223	M81223	Chicken	Germany	2298	1993
U65414.1	704	Chicken	Australia	2298	1996
L14767.1	L14767.1	Chicken	USA	2298	1999
AB027470.1	TR20	Chicken	Japan	2298	1999
AF313470.1	Del-Rosa ^a^	Chicken	USA	2294	2000
AB031296.1	A2	Chicken	Japan	2298	2000
AB046590.1	C369	Chicken	Japan	2298	2001
AF227982.1	AF227982	Chicken	Australia	2286	2001
AJ297685.2	clone34	Chicken	Germany	2297	2002
AF475908.1	AF475908	Chicken	China	2298	2002
AB119448.1	G6	Chicken	Japan	2298	2003
AY040632.1	3-IP60	Chicken	Malaysia	2298	2003
AF285882.1	SMSC-1	Chicken	Malaysia	2298	2003
AF390102.1	SMSC-IP60	Chicken	Malaysia	2298	2003
AY846844.1	TJBD40	Chicken	China	2298	2004
AF395114	BD-3	Chicken	Bangladesh	2298	2004
AY999018	SD24	Chicken	China	2298	2005
DQ124936.1	AH4	Chicken	China	2298	2005
AY839944.2	LF4	Chicken	China	2298	2005
DQ141673.1	SD22	Chicken	China	2298	2005
DQ124934.1	HA4	Chicken	China	2298	2005
DQ217401	SMSC-1P123WT	Chicken	Malaysia	2298	2005
DQ141671.1	SH16	Chicken	China	2298	2005
DQ141672	HN9	Chicken	China	2298	2005
JX964755	GXC060821	Chicken	China	2292	2006
EF683159	3711	Chicken	Australia	2279	2007
DQ991394	01-4201	Chicken	USA	2298	2007
KJ872513	CIAV-10	Chicken	Argentina	2298	2007
D10068.1	D10068.1	Chicken	Netherland	2298	2007
FJ172347.1	SDLY08	Chicken	China	2298	2008
D31965.1	82-2	Chicken	Japan	2319	2008
AF311892.2	98D02512	Chicken	USA	2298	2010
JF507715.1	CIAVV89-69	Chicken	Korea	2298	2011
KJ728827.1	18	Chicken	China	2298	2012
JX260426.1	GD-1-12	Chicken	China	2298	2012
KC414026	Cat-Gyv	Cat	China	2295	2012
JQ690762.1	JQ690762.1	Human	China	2316	2012
KM496308.1	SC-NC1	Chicken	China	2298	2013
KF224935.1	GD-K-12	Chicken	China	2298	2013
KM496307	SC-MZ42A	Chicken	China	2298	2013
KF224927.1	GD-C-12	Chicken	China	2298	2013
KM496306.1	SC-MZ	Chicken	China	2298	2013
KJ728824	14	Chicken	China	2298	2014
KU645520.1	HN1405	Chicken	China	2298	2014
KU645511.1	LN1402	Chicken	China	2298	2014
KY024579.1	RS-BR-15	Chicken	Brazil	2298	2015
KU645512.1	HN1504	Chicken	China	2298	2015
KU641014.1	JN1503	Chicken	China	2298	2015
KX811526.1	SD15	Chicken	China	2298	2015
KU645506.1	SD1512	Chicken	China	2298	2015
KY486137.1	HLJ15108	Chicken	China	2298	2015
KU645519	SD1508	Chicken	China	2298	2015
KU645510.1	SD1509	Chicken	China	2298	2015
KY486143.1	HLJ15169	Chicken	China	2298	2015
KU645524	CIAV-Dog	Dog	China	2298	2015
KU645525	CIAV-Mouse	Mouse	China	2298	2015
KX447636.1	LY-1	Chicken	China	2298	2016
KX447637.1	LY-2	Chicken	China	2298	2016
MN299312.1	1716TW	Chicken	China	2298	2016
MN299315.1	1535TW	Chicken	China	2298	2016
KX447634.1	BS-C2	Chicken	China	2298	2017
MG827100.1	CAV-SK4-2017	Chicken	Egypt	2298	2017
KX447633.1	BS-C1	Chicken	China	2298	2017
MK089243.1	17SY0902	Chicken	China	2298	2017
KX447635	HB160430	Chicken	China	2298	2017
NC001427	Cux-1 ^a^	Chicken	USA	2319	2018
MH001560.1	CAV-EG-13	Chicken	Egypt	2298	2018
KY486144.1	HLJ15170	Chicken	China	2298	2018
MK484615.1	GX1804	Chicken	China	2298	2019
MN103405.1	GX1805	Chicken	China	2298	2020
MW660821.1	SDSPF2020	Chicken	China	2298	2020
MN649256.1	GX1908L2	Chicken	China	2298	2021
MZ668716.1	HN2021-1414	Chicken	China	2298	2021
ON596886.1	Guangxi/2298/2021	Chicken	China	2298	2021
OL448839.1	SD2008	Chicken	China	2298	2021
OL448838.1	SD2007	Chicken	China	2298	2021
MZ540762.1	YN04	Chicken	China	2298	2022

^a^ Vaccine strain.

**Table 2:** Detailed information regarding the stains isolated in this study.

Accession No.	Strain Name	Species	Site of Isolation	Genome Length (bp)	Year
OQ869186	HN1901	Chicken	China	2298	2019
OQ869187	HB1901	Chicken	China	2298	2019
OQ869188	JS1901	Chicken	China	2298	2019
OQ869189	HN2001	Chicken	China	2298	2020
OQ869190	HB2001	Chicken	China	2298	2020
OQ869191	JS2001	Chicken	China	2298	2020
OQ869192	AH2001	Chicken	China	2298	2020
OQ869193	JS2002	Chicken	China	2298	2020
OQ869194	HN2002	Chicken	China	2298	2020
OQ869195	AH2002	Chicken	China	2298	2020
OQ869196	HB2101	Chicken	China	2298	2021
OQ869197	HB2102	Chicken	China	2298	2021
OQ869198	HN2101	Chicken	China	2298	2021
OQ869199	JS2101	Chicken	China	2298	2021
OQ869200	JS2102	Chicken	China	2298	2021
OQ869201	HN2102	Chicken	China	2298	2021
OQ869202	HB2103	Chicken	China	2298	2021
OQ869203	AH2101	Chicken	China	2298	2021
OQ869204	HB2201	Chicken	China	2298	2022
OQ869205	JS2201	Chicken	China	2298	2022
OQ869206	HN2201	Chicken	China	2298	2022
OQ869207	HN2202	Chicken	China	2298	2022
OQ869208	AH2201	Chicken	China	2298	2022
OQ869209	HN2203	Chicken	China	2298	2022
OQ869210	AH2202	Chicken	China	2298	2022
OQ869211	JS2202	Chicken	China	2298	2022
OQ869212	JS2203	Chicken	China	2298	2022

**Table 3:** Recombinant event I and related average *p*-values calculated using different recombination detection methods.

Methods	RDP	GENECONV	Bootscan	Maxchi	Chimaera	SiSscan	3Seq
*p*-Value	8.26 × 10^−7^	7.35 × 10^−6^	9.20 × 10^−6^	2.27 × 10^−9^	4.03 × 10^−10^	1.46 × 10^−15^	7.56 × 10^−17^

**Table 4:** Representative mutation sites of VP1 compared with the CIAV reference strain SD24.

Strain	Substitution of the Amino Acid Residues in VP1															
	22	75	89	97	125	139	141	144	157	287	290	370	376	394	413	446
SD24	H	V	T	M	L	K	Q	E	V	S	A	S	L	Q	A	G
HN1901	-	-	-	-	-	-	-	-	-	N	P	A	-	-	-	-
HB1901	-	-	-	-	-	-	-	-	M	-	-	G	I	-	S	-
JS1901	-	-	-	-	-	-	-	-	M	-	-	G	I	-	S	-
HN2001	-	I	-	L	I	Q	-	Q	-	-	-	G	I	-	S	-
HB2001	-	-	-	L	-	-	-	-	M	-	P	-	-	-	-	-
JS2001	-	-	-	-	-	-	-	-	-	-	-	-	M	-	-	-
AH2001	-	-	-	-	-	-	-	-	-	-	-	G	I	-	S	-
JS2002	-	-	-	-	-	-	-	-	-	-	-	G	I	-	S	-
HN2002	Q	-	-	L	-	-	-	-	M	T	P	-	-	-	-	-
AH2002	-	-	-	-	-	-	-	-	-	-	-	G	I	-	S	-
HB2101	-	I	-	L	I	Q	-	Q	-	T	P	T	-	-	-	-
HB2102	-	-	-	L	-	-	-	-	M	T	P	-	-	-	-	-
HN2101	-	-	-	-	-	-	-	-	M	-	-	G	I	-	S	-
JS2101	N	I	-	L	I	Q	-	Q	-	-	-	G	I	-	S	-
JS2102	-	-	-	-	-	-	-	-	-	-	-	G	I	-	S	-
HN2102	-	-	-	-	P	-	-	-	M	T	P	-	-	-	-	-
HB2103	N	I	-	L	I	Q	-	Q	-	A	P	G	I	-	S	-
AH2101	N	I	-	L	I	Q	-	Q	-	-	-	G	I	-	S	-
HB2201	-	-	-	-	-	-	-	-	M	-	-	G	I	-	S	-
JS2201	-	-	-	L	-	-	-	-	M	-	P	-	-	-	-	-
HN2201	-	-	-	-	-	-	-	-	M	-	-	G	I	-	S	-
HN2202	-	-	-	-	-	-	-	-	-	-	-	G	I	-	S	-
AH2201	-	-	-	L	-	-	-	-	M	T	P	-	-	-	-	-
HN2203	-	-	-	L	-	-	-	-	M	T	P	-	-	-	-	-
AH2202	-	-	-	-	-	-	-	-	-	-	-	G	I	-	S	-
JS2202	-	-	-	-	-	-	-	-	M	T	P	-	-	-	-	-
JS2203	-	-	-	-	-	-	-	-	M	T	P	R	-	-	-	-

## Data Availability

All the data generated or analyzed during this study are included in this article. Datasets are deposited in a publicly accessible repository; the datasets generated for this study can be found in GenBank: https://www.ncbi.nlm.nih.gov/Genbank/ (accessed on 18 August 2023). Genbank accession numbers are mentioned in the Materials and Methods of the article.

## Supplementary Materials

The following supporting information can be downloaded at: https://www.mdpi.com/article/10.3390/ani13172709/s1, Figure S1: A heat map of the similarity analysis of the entire genome of the CIAV strain and other reference strains obtained in this study (lower left: different identity values are expressed in gradient colors ranging from 91% to 100%).
