# Supplementary figures and images for: Molecular Characteristics of Chicken Infectious Anemia Virus in Central and Eastern China from 2020 to 2022
Source: Animals (Basel). 2023 Aug 25;13(17):2709. doi: 10.3390/ani13172709 (PMC10487239; doi:10.3390/ani13172709)

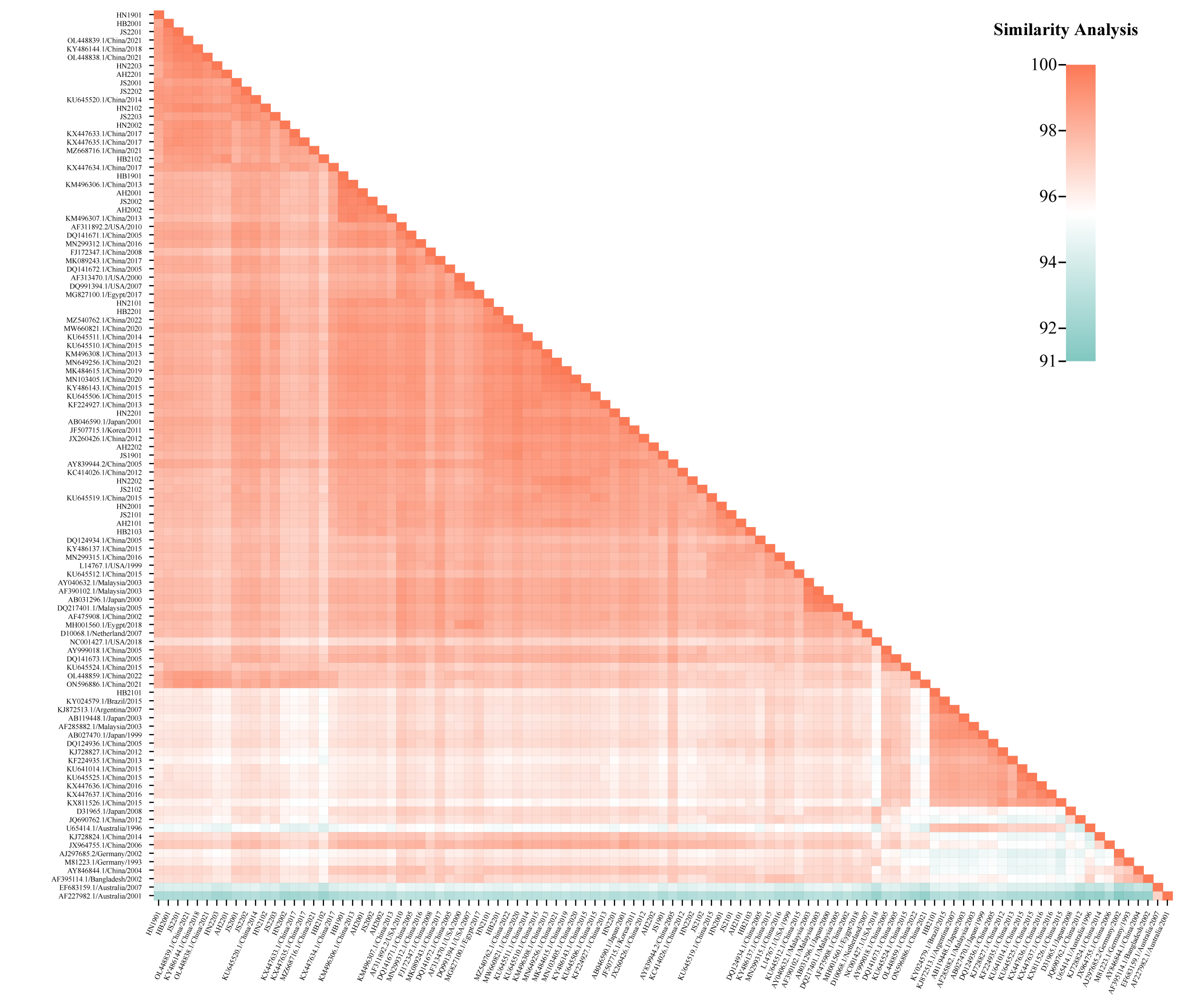

Supplement: Supplementary file 1 [file animals-13-02709-s001.zip › animals-2506618-Figure S1.tif]
